# Forecasting of excavation problems for high-rise building in Vietnam using planet optimization algorithm

**DOI:** 10.1038/s41598-021-03097-y

**Published:** 2021-12-10

**Authors:** Thanh Sang-To, Minh Hoang-Le, Samir Khatir, Seyedali Mirjalili, Magd Abdel Wahab, Thanh Cuong-Le

**Affiliations:** 1 Department of Electrical Energy, Metals, Mechanical Constructions and Systems, Faculty of Engineering and Architecture, Ghent University, Ghent, Belgium; 2 Faculty of Civil Engineering, Ho Chi Minh City Open University, Ho Chi Minh City, Viet Nam; 3 Centre for Artificial Intelligence Research and Optimisation, Torrens University Australia, Fortitude Valley, Brisbane, QLD 4006 Australia

**Keywords:** Civil engineering, Computational science

## Abstract

In this paper, a new method in forecasting the horizontal displacement of diaphragm wall (D.W.) for high-rise buildings is introduced. A new stochastic optimizer, called Planet Optimization Algorithm (P.O.A.), is employed to assess how proper finite element (F.E.) simulation is against field data. The process is adopted for a real phased excavation measured at the field. To automatically run the iterative optimization tasks, a source code is constructed directly in the Geotechnical Engineering Software (PLAXIS) by using Python to ensure that the operation between optimization algorithm and F.E. simulations are smooth to guarantee the accuracy of the complex calculation for the soil problem. The proposed process consists of two steps. (1) The parameters will be optimized at the early phases of the excavation. (2) The responses of D.W. displacements are forecasted at the subsequent phases. The aim of the process is to predict the displacements of D.W. of the building from the result of the nearby excavation or to provide early warning about the risks of excavation that may happen under vital phases. The proposed procedure also provides an effective method for optimization-based soil parameters updating in real engineering practice.

## Introduction

With the drastic development in the field of Computer Science in the past several decades, dealing with complex problems becomes easier by using a combination of Artificial Intelligence (A.I.) and finite element models^1^ to solve the problems. With growing challenges that require A.I. algorithms to be constantly improved^2–4^, many swarm-inspired algorithms such as Particle Swarm Optimization algorithm (P.S.O.)^5^, Genetic Algorithm (G.A.)^6^, or physics-inspired algorithm (e.g. Gravitational Search Algorithm^7^), even behavior-inspired of human (e.g. Human Behavior-Based Optimization^8^) have been proposed. Optimization algorithms are employed in most fields in daily life. In difficult and obscure fields such as soil and foundation mechanics, it is even more necessary to apply the strengths of both to maximize efficiency.

Skyscrapers have emerged as a solution to meet workplace or residence needs. While the used space expands upwards, the space inside the ground is also exploited for entertainment, storage, museums, or simply parking at the same time. Excavated construction for projects with two to four basements has become quite popular, in which the traditional method of digging with the anti-system has existed for a long time. This solution is simple and easy to run; it requires, nevertheless, a large area for construction.

Additionally, displacement of the soil and the diaphragm wall is usually large^9^. A semi-Top-Down construction solution is more suitable for excavation in urban space. This method has two advantages; short construction time and smaller displacement of the diaphragm wall than the braced excavation method.

Many previous studies show the influence of displacement of the diaphragm wall on adjacent buildings with the Semi-Top-Down (S.T.D.) constructed technique. Tan et al.^10^ studied the effects of subway station construction on surrounding buildings. Huang et al.^11^ presented a method combining S.T.D. excavation technique with steel bracing for the Shanghai's subway station. Peck^12^ proposed the inverse analysis or observational method. This technique is typically adopted to evaluate the dominant or representative characteristics or parameters of soil via observed data at the site.

Applying the nature-inspired optimization algorithm for the inverse analysis of the problem brings several benefits. This gives us a practical view of the suitable parameters for soil behavior.

One of the inverse analyses is a Bayesian updating procedure (e.g. Wang et al.^13^, Qi and Zhou^14^, Zhang and Mahadevan^15^, Hsiao et al.^16^ or Juang et al.^17^ employing Bayesian updating of soil parameters for prediction settlement or displacement of D.W., and Špačková and Straub^18^ using Bayesian for the tunnel excavation problem, etc.). Nevertheless, in the application of a model to update Bayesian, more than 10,000 calculations for the Markov chain of Monte Carlo sampling may be required, which makes the method impractical to use. In the last two decades, a new, more suitable method that has been widely used is the optimization algorithm. Specifically, it is particularly an intelligent algorithm to solving optimization problems. Yin and Jin^19^ demonstrated that an efficient multi-objective optimization-based updating framework could be constructed. Tang and Kung^20^ presented a study called a Nonlinear Optimization Method (NOM) to inversely analyze geotechnical engineering problems.

This study presents the model calibration's concepts based on inverse analysis. In the first section, the built methodology for the prediction of a horizontal deflection of diaphragm wall via an optimal combination of various design parameters of soil in the deep excavation problem is introduced. Specifically, a combination of F.E. using PLAXIS^21,22^ and programming language Python is constructed for inverse analysis of this problem. In which the objective function is defined by total displacement and settlement of excavation phases. Furthermore, soil parameters that are optimized at early phases of excavation are employed to predict the lateral deflection of D.W. and surface settlement at subsequent phases. Finally, the obtained results from model calibration are compared to field observation to verify the fitness of this technique and draw conclusions.

## Methodology

In deep excavation problems, settlement and displacement of the D.W. are the two important information on the construction of the structure of a high-rise building. In practice, the deformation ratio, which is defined as the maximum surface settlement over the maximum lateral displacement of D.W. introduced by Kung et al.^23^, can be adapted to predict the surface settlement profile based on the estimated maximum lateral displacement of D.W. This study, however, employs both displacement of the D.W. and settlement to enhance the efficiency and reliability of the model calibration. In this section, the proposed methodology is presented via three subsections, namely (1) simulation F.E. using PLAXIS, (2) novel optimization algorithm (P.O.A.), and (3) the link between (1) and (2) based on the environment of the Python language.

### Simulation model

#### Soil model

The geotechnical engineering software PLAXIS version 2020 is employed to calculate the behavior of the soil under the excavation. In more detail, the Hardening Soil Model (H.S.M.) is the soil model employed to characterize the soil in the PLAXIS simulation of the excavation. The stiffness of soil commonly is indicated by a set three modulus, namely the secant modulus in standard drained triaxial test ($E_{50}^{ref}$), the tangent modulus for primary oedometer loading ($E_{oed}^{ref}$) and Unloading/reloading modulus ($E_{ur}^{ref}$).

H.S.M. is a strong and modern model for the behavior of various soils, not only stiff soils but also soft soils^24^. A strong characteristic of the H.S.M. is the stress dependency of soil stiffness. For oedometer conditions of stress and strain, for instance, the model implies the relationship $E_{oed}^{{}} = E_{oed}^{ref} \left( {\sigma_{1}^{^{\prime}} /p^{ref} } \right)^{m}$. In many practical cases, it is appropriate to set $E_{ur}^{ref} = 3E_{50}^{ref}$. This is the default setting used in PLAXIS.

#### A case in Ho Chi Minh City

**Figure 1:**
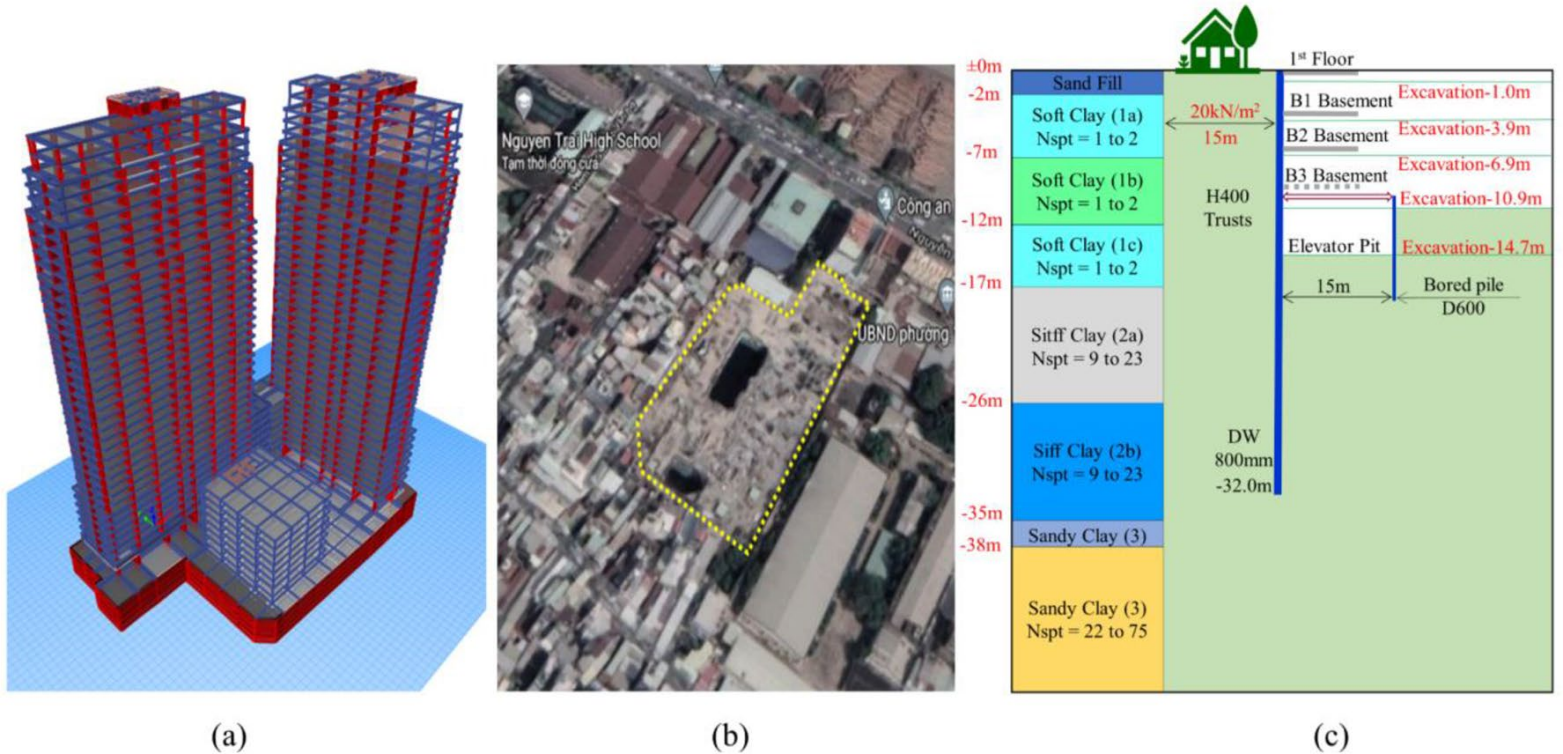
The Lancaster Tower project: (**a**) 3D view, (**b**) Nearby buildings of the project^25^, (**c**) Geotechnical data and main excavation stages.

Data for this problem is collected from the construction process of the Lancaster Lincoln Tower (L.L.T.), see Fig. 1a. This project is a proposed luxury residential and commercial development in Ho Chi Minh City, Vietnam. The development comprises two residential towers of 38 story’s, one office building of 7 story’s and three basements.

**Figure 2:**
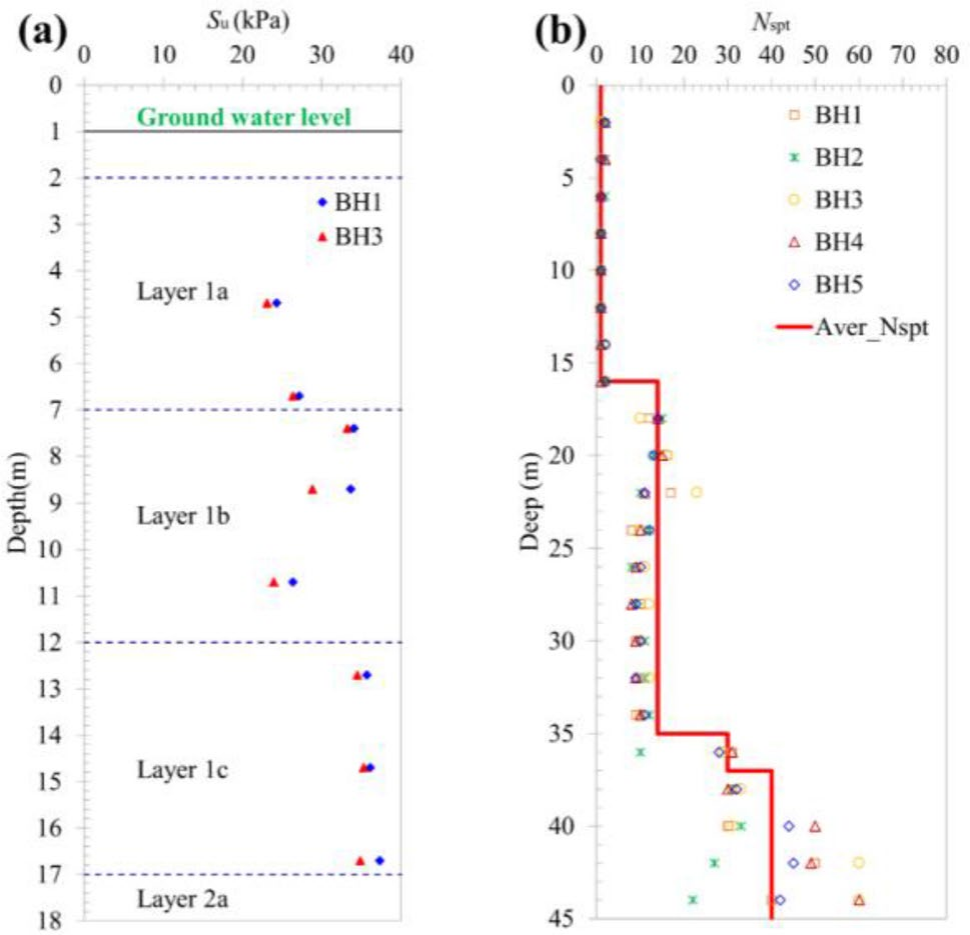
The parameters of soil from the investigation: (**a**) $S_{u}$ value (kPa), (**b**) $N_{spt}$ value.

**Figure 3:**
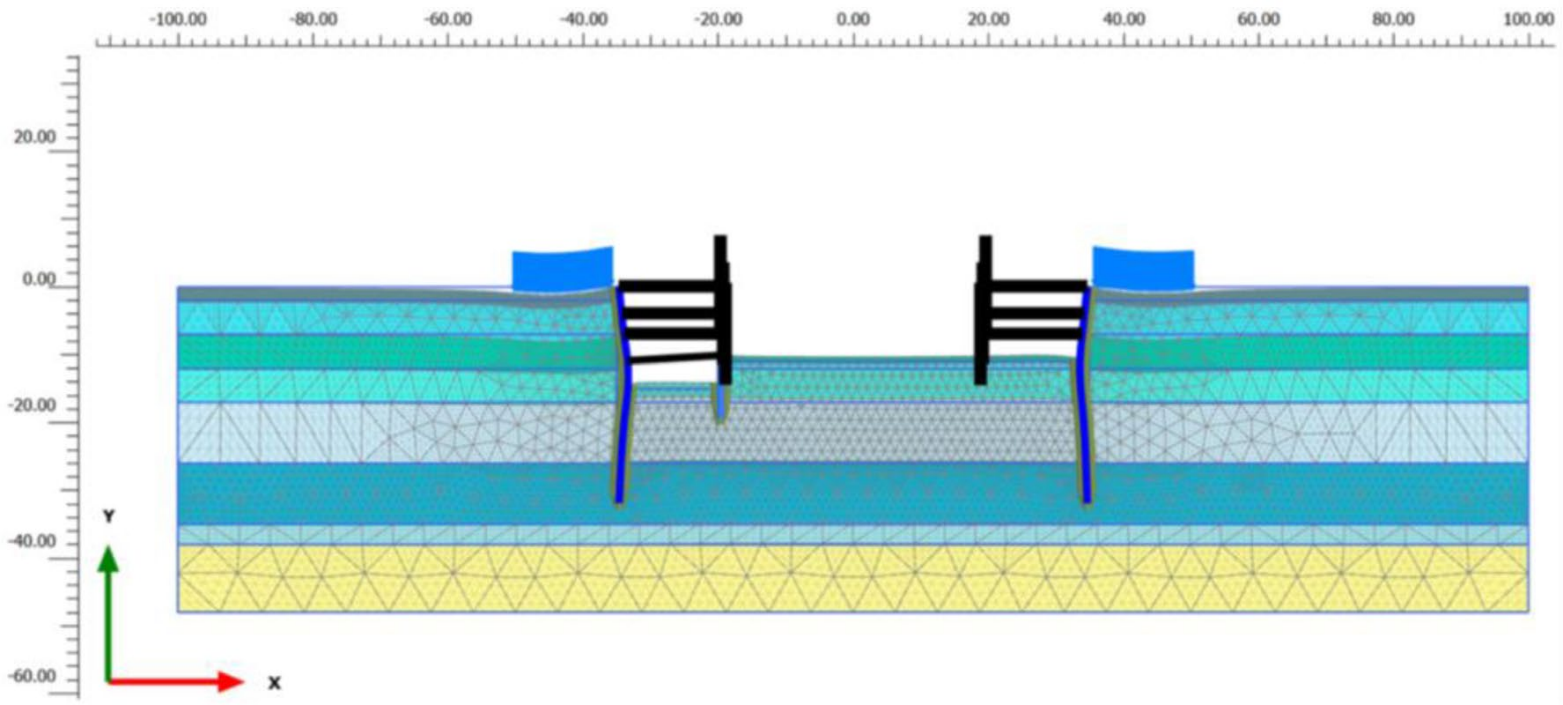
PLAXIS model for L.L.T. excavation.

The excavation width at the L.L.T. is 70 m, and the length of the 0.8 m thick diaphragm wall is 32 m with material properties: $EA$ = 2.02 × 10^7^ kN/m, $EI$ = 1,075,200 kNm^2^/m and Poisson’s ratio is 0.15. The excavation of Lancaster was performed employing the Semi -Top-Down technique. The excavation process underwent 5 main steps, in which the D.W. is supported by concrete slabs of 250 mm thick with material properties: $EA$ = 9.88 × 10^6^ kN/m and Poisson’s ratio is 0.15. The steps of excavation are summarized in Table 1. The geotechnical data of the project is illustrated in more detail in Table 2 and Fig. 2. The soft clay layer 15 m thick is divided into three layers, namely 1a, 1b, 1c whereas the stiff clay also is split into two sections, 2a and 2b as shown in the Fig. 1c. Meanwhile, Fig. 1b illustrates the adjacent buildings, one or two stories, for this reason, the surcharge load is chosen 20 kN/m^2^. Figure 3 illustrates the structural geometry and the PLAXIS mesh of the Lancaster excavation. The overall size of the model is 200 × 48 m^2^.

**Table 1:** Construction sequences for Lancaster Lincoln Tower.

Sequences	Construction activities
Stage 1	Excavation from existing to level-1.0 m
Stage 2	Excavation to level-3.9 m, construction of B1 slab
Stage 3	Excavation to level-6.9 m, construction of B2 slab
Stage 4	Excavation to level-10.9 m, construction of H400 trusts system
Stage 5	Excavation to level-14.7 m, level of elevator pit bottom

**Table 2:** Soil parameters of soil layers.

No	Soil	Type	*γ* (kN/m^3^)	*m*	*ν*	*S*_u_ (kN/m^2^)	*c'* (kN/m^2^)	*φ'* (°)	*ψ*(°)	*R*_inter_
0	Sand fill	Drained	18.0	0.50	0.2	–	5	30	–	0.5
1a	Soft clay	Undrained	14.8	0.90	0.2	23.1 ~ 26.4	–	–	–	0.8
1b	Soft clay	Undrained	14.8	0.90	0.2	23.9 ~ 33.3	–	–	–	0.8
1c	Soft clay	Undrained	14.8	0.90	0.2	34.5 ~ 35.3	–	–	–	0.8
2a	Stiff clay	Undrained	19.2	0.75	0.2	70 ~ 110	–	–	–	0.8
2b	Stiff clay	Undrained	19.2	0.75	0.2	110 ~ 150	–	–	–	0.8
3	Clay sand	Drained	20.2	0.50	0.2	–	10.2	22.3	–	0.8
4	Fine sand	Drained	20.9	0.50	0.2	–	3.9	33.5	3.5	0.8

While the three first excavation stages are employed to search soil parameters by using PLAXIS and Python, the lateral displacement of D.W. and settlement under two final-stages excavation are predicted based on these optimized parameters at early-phases.

#### Soil parameter for inverse analysis

In recent decades, many researchers^26–28^ tried to determine the stiffness and strength parameters for H.S.M. Some scholars made a concentrated effort to back calculate the ratio of the stiffness modulus of the undrained shear strength ($E/S_{u}$). The undrained shear strength $S_{u}$ is evaluated by the vane shear tests in soft clay or triaxial tests on stiffer clay. These tests are normally employed in the inverse analyses of the $E/S_{u}$ ratio. Several studied results in the world are summarized in Table 3.

**Table 3:** Results of $E/S_{u}$ ratio from F.E. inverse analyses.

	Tang and Kung^20^	Hsiung et al.^29^	Phienwej and Gan^30^	Likitlersuang et al.^31^
Very soft clay	300–700	500	500	500
Stiff clay	700–2000		1200	600–1000

Meanwhile, $E^{\prime}$(stiffness parameter) is employed to indicate the stress–strain behavior of sandy soil. However, many physical soil characteristics (e.g. structure, location of the sand grains into sample, which is affected significantly by the disturbance due to the process sample collection at the field) can have a powerful influence on stiffness parameter. In practice, $E^{\prime}$ can be computed using the results of the Standard Penetration Test with the $N_{spt}$ value. Hsiung et al.^29,32,33^ proposed an empirical correlation between $N_{spt}$ and stiffness parameter as follows $E^{\prime} = (2000 - 4000) \times N_{spt}$.

In this study, stiffness parameters are employed for updating the optimization framework. While many studies estimated secant modulus in [300, 700] $S_{u}$ for very soft clay and [500, 1200] $S_{u}$ for stiff clay, the proposed method randomly selects an initial value in the range [200, 700] $S_{u}$ for very soft clay and [400, 1000] $S_{u}$ for stiff clay. Meanwhile, this stiffness parameter value is [2000, 4000] $N_{spt}$ for sandy layers. In other words, the search space is larger than some normal results to evaluate the operation of this optimization process. Table 4 illustrates in detail the soil parameters for each soil layer. In which *low* and *up* are lower and upper bounds of each parameter, respectively.

**Table 4:** Parameters of soil and boundary for input data PLAXIS-Python.

Type		Soft clay	Soft clay	Soft clay	Stiff clay	Stiff clay	Clay sand	Fine sand
		Layer 1a	Layer 1b	Layer 1c	Layer 2a	Layer 2b	Layer 3	Layer 4
		Un-Drained	Un-Drained	Un-Drained	Un-Drained	Un-Drained	Drained	Drained
$S_{u}$ kN/m^2^		23.1–26.4	23.9–33.3	34.5–35.3	70–110	110–150	–	–
$N_{spt}$		1–2	1–2	1–2	9–23	9–23	10–31	22–60
$E_{50}^{ref}$ kN/m^2^	low	5000	5740	6894	36,000	52,000	60,000	80,000
	up	18,000	20,090	24,129	90,000	130,000	120,000	160,000

### Optimization model

Using the optimization method is vital for finding optimal solutions for different optimization problems. In this subsection, we present a summary of the new Planet Optimization Algorithm (P.O.A.). The P.O.A. theory will be fully developed in the other paper, and consequently is not reproduced here. In the paper, a brief description of the algorithm is, however, also presented including flowchart.

The basic concept of gravitation laws (or Newton's law) will be presented. Next, inspired by the motion of planet in the universe by this law, a mathematical model for an optimal algorithm will be constructed.

1 $$F = G \times \frac{MassA \times MassB}{{\left\| {R_{{_{AB} }}^{{}} } \right\|^{2} }}$$

where $F$ is the gravitational force, $G$ is the gravitational constant, $R_{AB}$ is the distance between the centers of their masses, $MassA,MassB$ are the mass of each planet.

In this study, the parameter $F$ is ineffective in conducting the search process of the algorithm. Thus, moment force $M = F \times R$ is adopted to operate as the main parameter instead of $F$.

**Figure 4:**
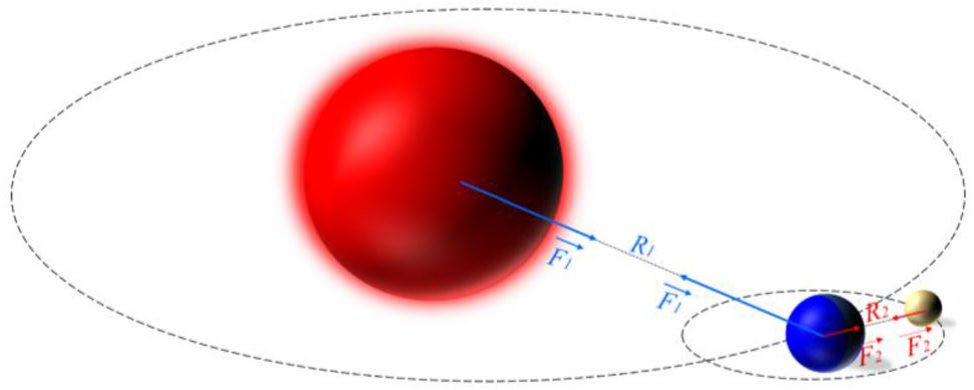
The interaction of three planets for Newton's law.

The optimized model is illustrated simply by a system consists of the Sun, the Earth and the Moon as shown in the Fig. 4. In more detail, each object is impacted by two forces. For instance, for the Earth, 2-force are from the sun and the Moon. The Earth can create a gravitational force large enough to keep the moon in orbit around the Earth. This demonstrates that two parameters influence the motion of a planet, not only mass but also the distance between the two planets. Specifically, the implementation of the algorithm is as follows:

**Stages 1: The best start.** The algorithm's first step is to search for an effective solution to operate as the best solution. The aim of this step is to improve the convergence and accuracy in the first iterations.

**Stages 2: Calculation **$M$** factor.**

2 $$\left| {\overrightarrow {M} } \right| = \left| {\overrightarrow {F} } \right| \times \left\| {R_{{{\text{ij}}}}^{{}} } \right\| = G\frac{{mass_{i} \times mass_{j} }}{{\left\| {R_{{{\text{ij}}}}^{{}} } \right\|^{2} }} \times \left\| {R_{{{\text{ij}}}}^{{}} } \right\|$$

where, $$mass_{i} ,mass_{j} = \frac{1}{{a^{{{\raise0.7ex\hbox{${obj_{i,j} }$} \!\mathord{\left/ {\vphantom {{obj_{i,j} } \alpha }}\right.\kern-\nulldelimiterspace} \!\lower0.7ex\hbox{$\alpha $}}}} }}$$;$$a = 2;\alpha = \left| {\max (obj) - obj_{sun} } \right|$$; $$obj_{i,j} ,\max (obj),obj_{sun}$$ are the value of objective function of the *i*^th^ or *j*^th^ planet, the worst planet and the sun, respectively. This means that the objective function value of a planet is smaller, the mass of this planet is larger. The distance $$\left\| {R_{{{\text{ij}}}} } \right\|$$ is the Cartesian distance between two planets *i* and *j* at $$X_{i}^{t}$$ and $$X_{j}^{t}$$ , respectively. Meanwhile, $$G$$ is a constant and equal to one unit in this algorithm.

3 $$\left\| {R_{{{\text{ij}}}} } \right\| = \left\| {X_{i}^{t} - X_{j}^{t} } \right\| = \sqrt {\sum\limits_{k = 1}^{k = \dim } {\left( {X_{i}^{t} - X_{j}^{t} } \right)^{2} } }$$

**Stages 3: Global search. **$\beta$ = *M*_*i*_/*M*_max_ is a coefficient, which depends on $M$ moment presented in Eq. (2). In which *M*_*i*_ is the sun's gravity on a plane*t i*^th^ at *t* iteration, and $M_{\max }$ is the value of $\max \left( {M_{i}^{t} } \right)$ at $t$ iteration. The $\beta$ coefficient, therefore, contains values in (0, 1).

4 $$X_{i}^{t + 1} = X_{i}^{t} + b \times \beta \times r_{1} \times \left( {X_{Sun}^{t} - X_{i}^{t} } \right)$$

**Stages 4: Local search.** When the distance between the Sun and a planet is small, the local search process is operated. The planet with the biggest mass will operate like the sun. It means that the planet moves a small distance between it and the hypothetical Sun at iteration t instead of going straight towards the hypothetical Sun. This is illustrated and Eq. (5).

5 $$X_{i}^{t + 1} = X_{i}^{t} + c \times r_{1} \times \left( {r_{2} \times X_{sun}^{t} - X_{i}^{t} } \right)$$

where $c = c_{0} - t/T; \, c_{0} = 2$, with $T$ is the maximum number of iterations. $r_{1}$ is a chaotic function in (0,1) whereas $r_{2}$ is normal distribution function (mean value $\mu = 0.5$ and standard deviation $\sigma = 0.2$).

**Figure 5:**
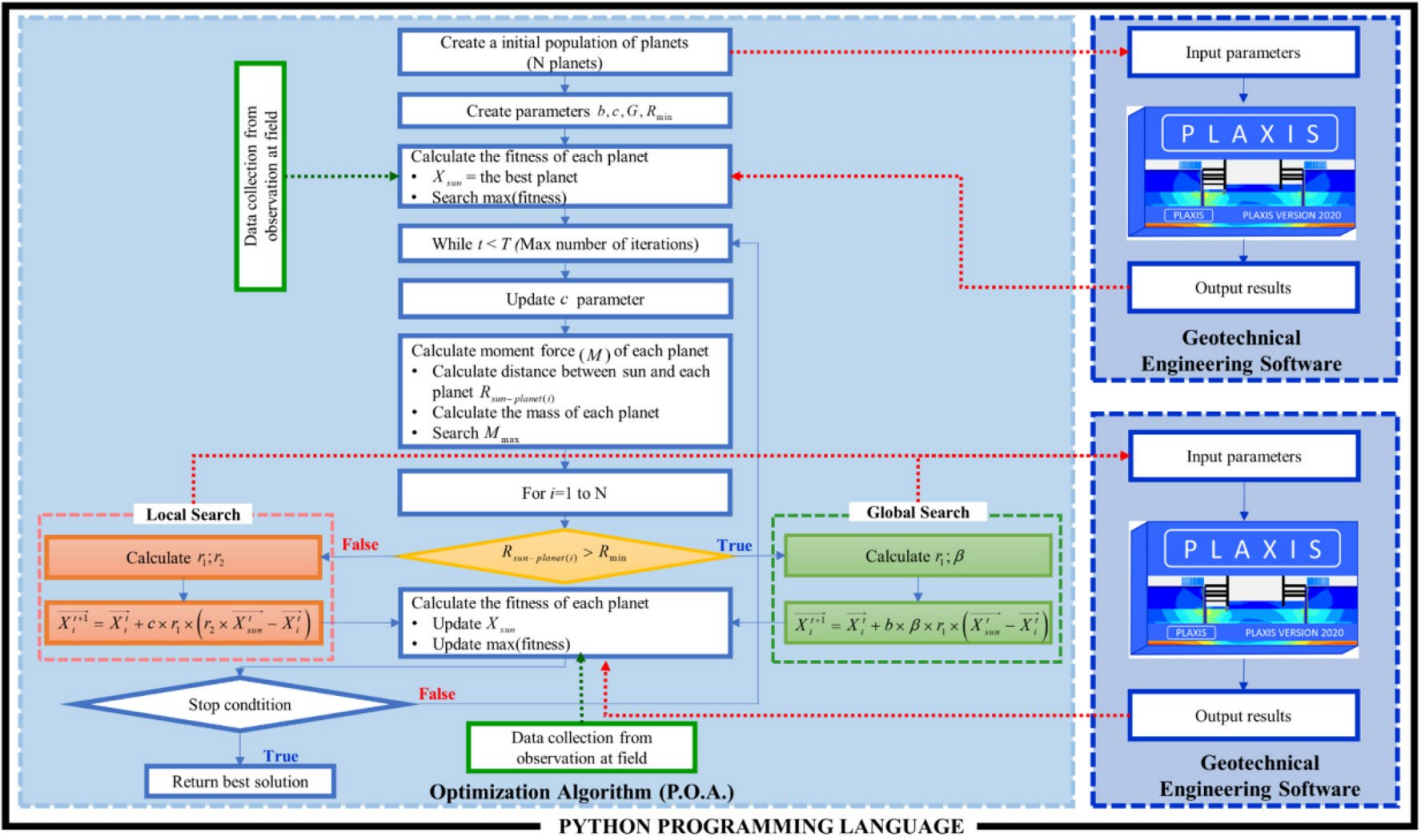
Schematic diagram of the proposed methodology.

In sum, a flow chart is proposed for the new planet optimization algorithm (P.O.A.) summarized in the Fig. 5. Where,$R_{\min }$ is chosen by dividing the search space into 1000-zone as shown Eq. (6).

6 $$R_{\min } = \frac{{\left\| {up - low} \right\|}}{{R_{0} }}$$

where *low* is lower bounds, and *up* is upper bounds of the problem; $R_{0} = 1000$**.**

### Combined simulation–optimization framework

The meta-heuristic optimization algorithm (P.O.A.), which is integrated into geotechnical engineering software, is adopted to solve this problem. Each set of parameter values is sent to the simulation model using PLAXIS, which has been calibrated for the particular soil system. Then, the simulation model is solved to obtain the resulting response in the form of displacements of diaphragm wall at phases. This displacements values and observed data at field are utilized by P.O.A. to solve the objective functions $\left( {obj} \right)$ as shown in Eq. (7). With the Python environment, a strong and fast connection is created between PLAXIS and the optimization algorithm. It means that the P.O.A. is easy to proceed with the information from PLAXIS accurately without any interruption. This proposed procedure is iterated until the specified termination criterion is reached. The developed methodology for the estimation displacement of the diaphragm wall is presented with a schematic diagram in Fig. 5.

7 $$obj = w_{d} \times \frac{{\sum\limits_{i = 1}^{{n_{d} }} {\left( {DOF_{i} - DSP_{i} } \right)^{2} } }}{{n_{d} \times N_{p} }} + w_{s} \times \frac{{\sum\limits_{j = 1}^{{n_{s} }} {\left( {SOF_{i} - SSP_{i} } \right)^{2} } }}{{n_{s} \times N_{p} }}$$

- $DSP_{i}$ is a point's displacement of D.W. simulated by F.E. in PLAXIS at phase *i*th.
- $DOF_{i}$ is a point's displacement of D.W. obtained from field observation at phase *i*th.
- $SSP_{i}$ is a point's settlement simulated by F.E. in PLAXIS at phase *i*th of the adjacent building.
- $SOF_{i}$ is a point's settlement obtained from field observation at phase *i*th of the adjacent building.
- $n_{d} ,n_{s} ,N_{p}$ are the observed node number of D.W., settlement and phases, respectively.
- $$w_{d} = 1$$, $$w_{s} = 0.5$$ are weights.

Up to sum, this methodology that combine of F.E. method using PLAXIS and the optimization algorithm (P.O.A.), in which Python language is operated as a strong connection both together.

## Results and discussion

### Comparison P.O.A., G.A. and P.S.O.

**Figure 6:**
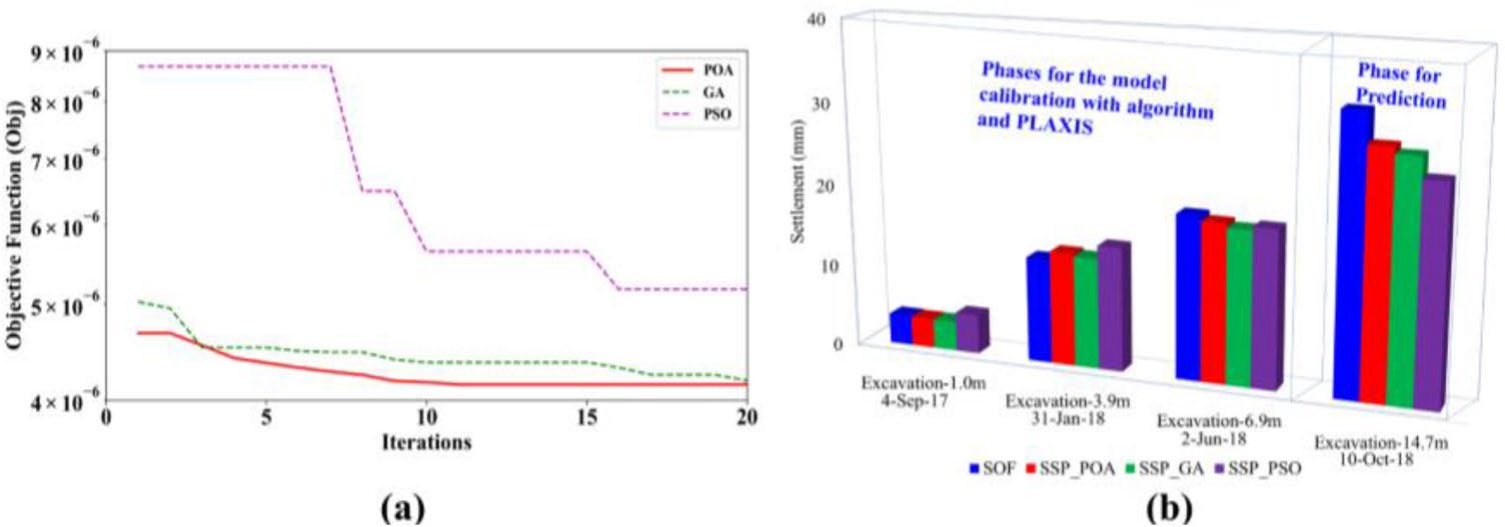
Results of comparison between P.O.A and candidates: (**a**) Convergence curve (best solution in each iteration), (**b**) Monitored settlement of the near building.

**Figure 7:**
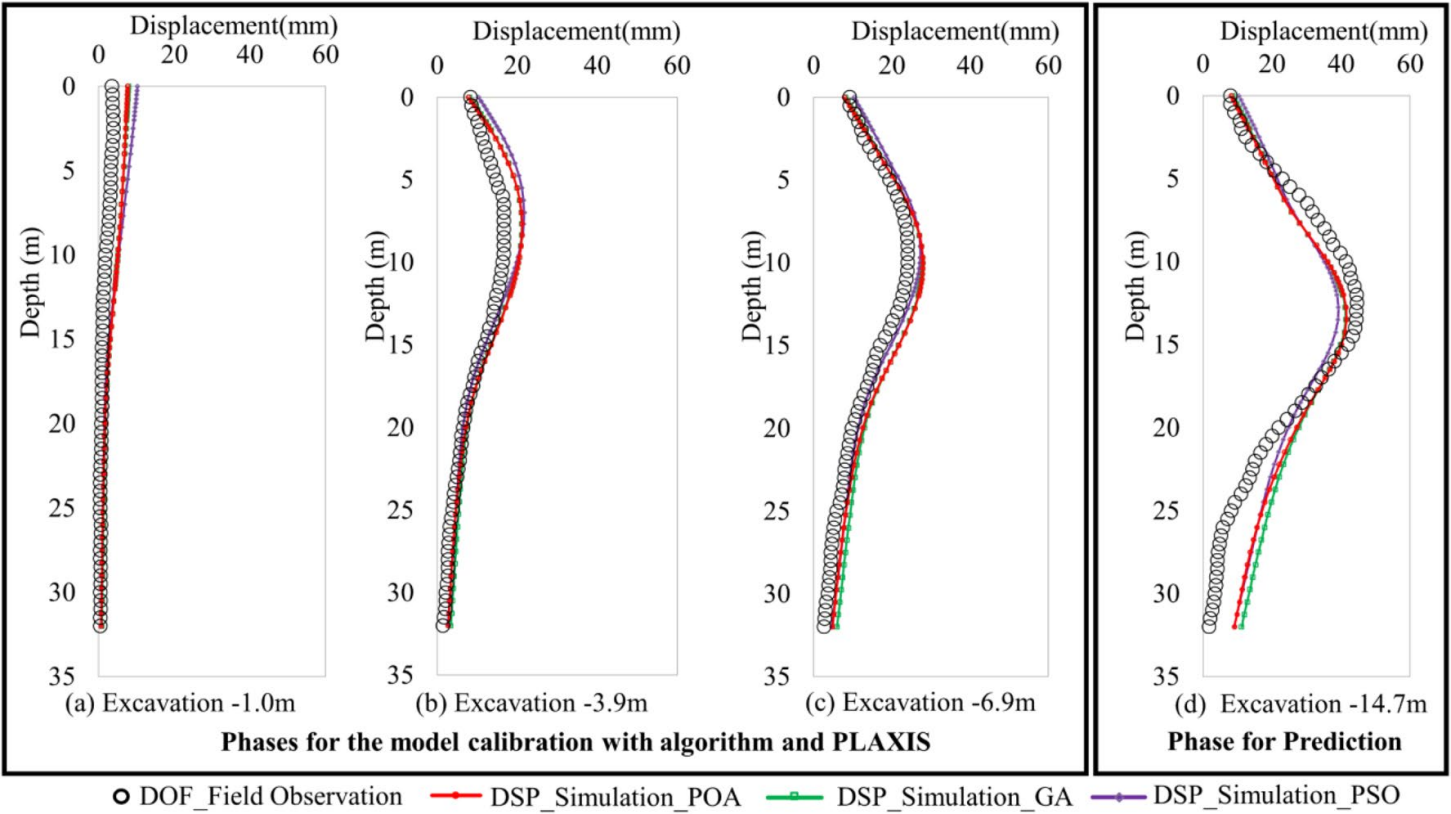
D.W. displacements.

In this subsection, the comparison between P.O.A. and two well-known algorithms (P.S.O. and G.A.) for forecasting real excavation problems is presented. A thorough investigation is conducted for comparison between computed displacements and field data via observations from inclinometers of the excavation. The results of the P.O.A. are presented for comparison with the results of the G.A. and P.S.O. in this problem. It means that the proposed methodology replaces P.O.A. with G.A. or P.S.O. Furthermore, by doing that, we can evaluate the effect of optimization algorithm on the proposed method, check the performance of P.O.A. at the same time. Figure 6a shows the value of the objective function in predicting D.W. displacement at subsequent phase via the soil characteristics inverse analysis at early phases. For example, the information about the displacement of points indicated in Fig. 7d represents the predictions of the D.W. displacements at the final phase of excavation, stage 5, employing the soil parameters inverse analysis at the stages of 1–3, respectively, based on the observed D.W. displacements at the corresponding phases. It can seem that the objective function of algorithms is quite small, by doing that, it demonstrated that the method is effective. However, how good the effect of the method is depending on the superiority of the optimal algorithm in this problem. The results of P.O.A. is more accurate than G.A. and much outperform P.S.O. As we can see in Fig. 6a, P.O.A. takes only 11 iterations for finding out the best solution, while G.A. and P.S.O. cannot converge.

Figure 6b presents the comparison of surface settlement between predictions and field data by the bar chart. It is apparent that the prediction of surface settlement by P.O.A. is acceptable and approximately identical between field observation and prediction with simulation. To some extent, this prediction result shows that P.O.A. is achieving better value than P.S.O. and G.A. in the final stage of excavation.

The inverse analysis D.W. displacements of each excavation phase in the L.L.T. are indicated in Fig. 7a–c. In this case, the horizontal displacement of D.W. is significantly influenced by stiffness of layer 1 and layer 2. Clearly, the results of the model calibration by P.O.A. and G.A. are closely similar in first-stage excavation, because both algorithms explore the proper modulus $E_{50}^{ref}$ of layer 1 (see Table 5). There is a similarity in the ${{E_{50}^{ref} } \mathord{\left/ {\vphantom {{E_{50}^{ref} } {S_{u} }}} \right. \kern-\nulldelimiterspace} {S_{u} }}$ ratio of 1a, 1b and 1c layers, which means that P.O.A. and G.A. verify three layers had the same physical properties, in other words, they are one soil layer. With P.S.O. it is the other way around, this ratio is the large difference between layer 1a and 1c. Which leads to the results of displacement D.W. updated by PSO is a substantially discrepancy in comparison with the others. It is interesting to note that all candidates provide good results of ${{E_{50}^{ref} } \mathord{\left/ {\vphantom {{E_{50}^{ref} } {S_{u} }}} \right. \kern-\nulldelimiterspace} {S_{u} }}$ ratio for the layer 2. Nevertheless, once again, we can see that the results of P.O.A. are more suitable than other algorithms, because the displacement of the D.W. and the ground settlement based on the analysis using P.O.A. are closer to reaching the field observations than G.A. or P.S.O. The above reasons illuminate why target parameters of P.O.A. are the more proper. Consequently, the objective function value of P.O.A. far outstanding to G.A. and P.S.O.

**Table 5:** ${{E_{50}^{ref} } \mathord{\left/ {\vphantom {{E_{50}^{ref} } {S_{u} }}} \right. \kern-\nulldelimiterspace} {S_{u} }}$ and ${{E^{\prime}} \mathord{\left/ {\vphantom {{E^{\prime}} {N_{spt} }}} \right. \kern-\nulldelimiterspace} {N_{spt} }}$ ratio for soil layers.

Parameter	Soil Layer	Depth	Optimization algorithm		
		(m)	P.O.A	G.A	P.S.O
${{E_{50}^{ref} } \mathord{\left/ {\vphantom {{E_{50}^{ref} } {S_{u} }}} \right. \kern-\nulldelimiterspace} {S_{u} }}$	layer1a	2 ~ 7	458	457	298
	layer1b	7 ~ 12	479	475	357
	layer1c	12 ~ 17	402	417	675
	layer2a	17 ~ 26	628	674	778
	layer2b	26 ~ 35	747	564	582
${{E^{\prime}} \mathord{\left/ {\vphantom {{E^{\prime}} {N_{spt} }}} \right. \kern-\nulldelimiterspace} {N_{spt} }}$	layer3	35 ~ 38	2534	2041	3469
	layer4	38 ~ 48	2411	2000	3691

Up to the sum, the P.O.A. provides the prediction of horizontal displacements of retaining wall is more suitable than G.A. and P.S.O. Although the difference of value of displacement D.W. is not large between algorithms, it also verifies that P.O.A. explores effectively and strongly in optimization.

### Discussion about results of the proposed methodology using P.O.A.

**Figure 8:**
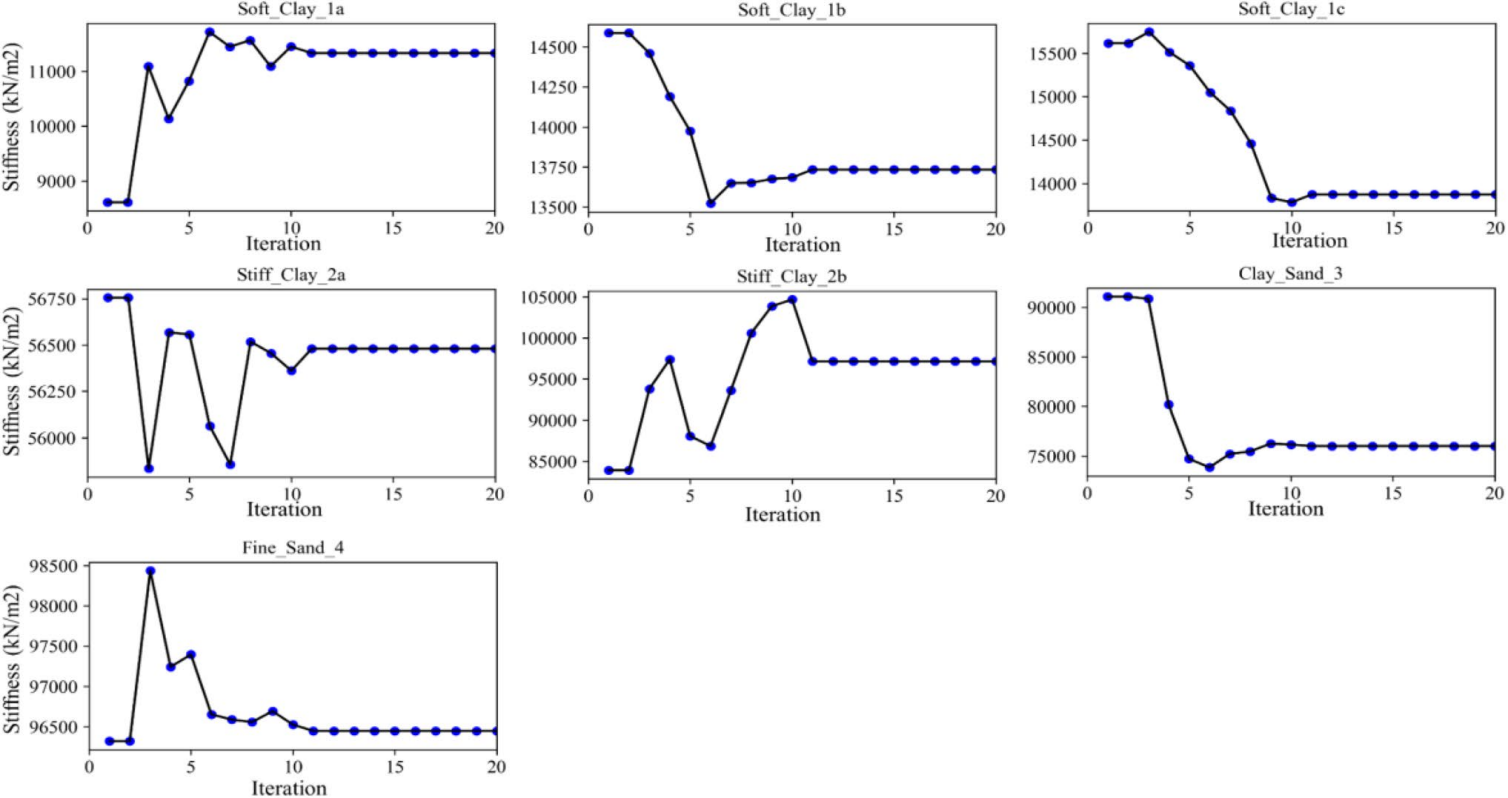
The trajectory of the best planet (the value of $E_{50}^{ref} - kN/m^{2}$ for each soil layer) when back-figured parameters based on three early-stage excavations by P.O.A.

In practice, the inverse analysis combination with excavation is to make a forecast the final soil behavior of excavation on what happened in early-phase observations. First, the target parameters have to be optimized at the early phases of the excavation. Next, the responses of D.W. displacement at the subsequent phases, especially the final phase of excavation, are predicted under target parameters optimized at early phases. Consequently, the results of inverse analysis of the L.L.T. is adopted to further experiment the capacity of the developed method. The ${{E_{50}^{ref} } \mathord{\left/ {\vphantom {{E_{50}^{ref} } {S_{u} }}} \right. \kern-\nulldelimiterspace} {S_{u} }}$ ratio in the range [400, 750] inverse analysis of the soils, including [400,500] for soft clay (1a, 1b and 1c) and [600, 750] for stiff clay (2a and 2b), which are considered the proper previous researches^20,29,31–33^. The back-figured values of modulus for both layer 3 and 4 in [2400, 2600] are essentially suitable for recommendation of researchers^29,32,33^. The identification iteration paths illustrated in Fig. 8 reflect how the variation of updating parameters with excavations gradually converges to reach the real parameters.

In this case, the inverse analysis is employed for only one target parameter for each soil layer, however, this back computing provides the essential accuracy and satisfaction for inverse analysis D.W. displacements. It is clear that for back-figured parameters for D.W. displacement in early-stage excavation are approximately identical with field observation despite the differences. This is acceptable in real geotechnical engineering problems because these errors are quite small. Additionally, the shape of the displacement line of D.W. is very proper with field monitoring data. Furthermore, these errors at the maximum displacement points do not exceed 3 mm, which are highly desirable results in the design field of real geotechnical engineering problems. Generally, the soil parameters inverse analysis at early phases can be employed to predict the maximum D.W. displacements at stage 5 satisfactorily. It means that the similar soil parameters inverse analysis at different phases may be representative and is approaching the real soil parameters.

## Conclusions

Based on the analyses and comparisons presented herein, the following conclusions can be drawn:

(1) The inverse analysis procedure, which is a fully automated process integrated the optimization algorithm (P.O.A.) with the Python code into geotechnical software (PLAXIS) platform, is effectively employed for the model calibration of lateral movements at the excavation. Simultaneously, it is also employed in conjunction with field observations to increase practicality. (2) From early observations about lateral movements of D.W. and surface settlement in the first excavation, stages are employed to recalibrate the model, which can "adequately" estimate the responses of the soil for the subsequent excavation stages. This method is significant in that a successfully updated model at the first excavation stages, which affects all processes, is the automatic forecast of the soil behaviour whole excavation. (3) The growing accuracy of the computed results of the objective function and relative fit enhancement are statistics that proof this technique's efficiency.

With the inverse analysis method based on monitored data in the field at the early phases of excavation or from nearby excavation buildings, model parameters may be obtained easily and reliably instead of the traditional approaches, which depend mainly on the pure experience of engineers. Forecasting accidents due to extraordinary displacement D.W. and the surface settlement in time provides a good risk forecasting guarantee for the proper prevention. This research provides a comprehensive methodology for predicting risk, enhancing safety, saving time and budget, and effectively designing and constructing high-rise buildings. Furthermore, with the drastic development of the Computer Science field and Artificial Intelligence (A.I.) in recent decades, we firmly believe that the technique will be trending in the future inevitably.
